# Supplementary material for: Needs assessment of a pythiosis continuing professional development program
Source: PLoS Negl Trop Dis. 2024 Feb 26;18(2):e0012004. doi: 10.1371/journal.pntd.0012004 (PMC10919846; doi:10.1371/journal.pntd.0012004)
Supplement: S1 Supplement — Survey for evaluating the necessity of developing the vascular pythiosis training program. (DOCX) [file pntd.0012004.s001.docx]

**Survey for Evaluating the Necessity
of Developing the Vascular Pythiosis Training Program.**

**Instruction:** Respondents are free to answer the questions voluntarily. The responses to this questionnaire will be anonymous, without any personal identification, and will not have any impact on the evaluation of the residency training program. You can provide information by circle the most valid answer.

**Part 1: basic information of respondents**

1. What is your gender?

A. Male B. Female

1. What is your residency training program?

A. Internal medicine B. Surgery

1. What is your postgraduate year?
   A. Year 1 B. Year 2

C. Year 3 D. Year 4 E. Year 5

**Part 2: test of knowledge on vascular pythiosis**

1. In which country are the highest number of reports of vascular pythiosis in human?
   a. Thailand b. India c. Brazil d. Australia e. United States
2. Vascular pythiosis in Thailand are most commonly found in which region?

a. Northern Region b. Central Region c. Northeastern Region
d. Choices a, b e. Choices a, b, and c

1. Which disease is most commonly associated with vascular pythiosis patients?

a. Lupus (SLE) b. Diabetes
c. Colorectal cancer d. Hemolglobinopathy
e. Interferon-gamma autoantibody immunodeficiency

1. Which occupation is the most significant risk factor for contracting vascular pythiosis?

a. Veterinarian b. Farmer c. Fruit vendor
d. Bus fare collector e. Blacksmith

1. Which of the following is a physical examination finding typically seen in patients with vascular pythiosis?

a. Chronic ulcers b. Enlarged lymph nodes
c. Palpable mass at vessel area
d. Absent or decreased pulse in peripheral artery e. All of the above

1. In a 20-year-old Thai male patient with a chronic ulcer on the right shin for one month, no fever, and no improvement with dicloxacillin treatment, which of the following findings supports the diagnosis of vascular Pythiosis?
   a. Inflammatory, red, and pus-filled ulcers
   b. Enlargement of inguinal lymph nodes
   c. Erythema nodosum-like lesion
   d. Absence of the right popliteal artery pulse
   e. Sporotrichoid lesions (lymphocutaneous spreading)
2. Which symptoms is associated with vascular claudication in patients with vascular pythiosis?

a. Muscle weakness b. Pain when climbing stairs
c. Gradual worsening of symptoms when climbing stairs
d. Rapid deterioration of symptoms when descending stairs
e. Improvement of symptoms when bending forward or squatting

1. A 30-year-old Thai female patient with an ulcer on the right leg for one month, Which of the following is **NOT** consistent with the symptoms of vascular pythiosis?

a. Occasional low-grade fever
b. Weakness in the right leg muscles
c. Dry, non-pus-producing ulcer
d. Firm lump at the right knee
e. Lack of response to beta-lactam antibiotics

1. What is the natural source of the Pythium insidiosum?

a. Flooded areas with communities b. Industrial factory water sources
c. Water sources related to agriculture d. Constantly flowing water
e. Flooded areas following natural disasters

1. A 45-year-old Thai male patient has been diagnosed with vascular pythiosis. In this case, the infection occurred through contact with which infective form?
   a. Hyphae b. Oospores c. Zoospores d. Endospores e. Blastospores
2. Which type of sample collection and transport is most suitable when the sample transport time exceeds 12 hours for diagnosing vascular pythiosis?

a. Transporting an arterial wall sample in a sterile container without freezing
b. Transporting a blood clot sample in artery in a sterile container with freezing
c. Transporting a skin sample from the area with a color change in a sterile container without freezing
d. Transporting a whole blood sample in a freezing and sterile tube
e. Transporting a plasma/serum sample in a freezing and sterile tube

1. A 40-year-old male patient was found to have a chronic wound on his right leg. Which laboratory test can help confirm the diagnosis of vascular pythiosis?

a. Positive of biochemical analysis of (1,3)-Beta-D-Glucan in the patient's blood sample.
b. Specific antibodies to P. insidiosum were detected in the patient's blood sample.
c. KOH Preparation revealed the presence of rare Septate Hyphae in the arterial walls.
d. Cultivation of the fungus from a blood clot in the patient's artery showed submerged Hyphae Colony.
e. Genetic material of the fungus was detected using Polymerase Chain Reaction (PCR) in the blood clot obtained from the patient's artery."

1. To assess the severity of the vascular pythiosis and its extent, which radiological examination is the most suitable option?

a. Ultrasound b. PET/CT scan c. Plain radiograph

d. Plain MDCT scan e. Contrast enhanced MDCT scan

1. A 42-year-old male patient has had a chronic wound on his left leg for 6 weeks. He has a history of stage 4 chronic kidney disease. If you need to perform a radiological examination to assess the severity of the disease, which examination is the most appropriate choice?

a. PET/CT scan b. Plain radiograph c. Plain MDCT scan
d. Non-contrast enhanced MRA e. Angiogram using CO_2_ as contrast agent

1. In patients with vascular pythiosis, various types of vascular abnormalities can be found. Which one is the least commonly found?

a. aneurysm b. arteritis or phlebitis c. arterial occlusion

d. atherosclerosis e. ruptured pseudoaneurysm

1.
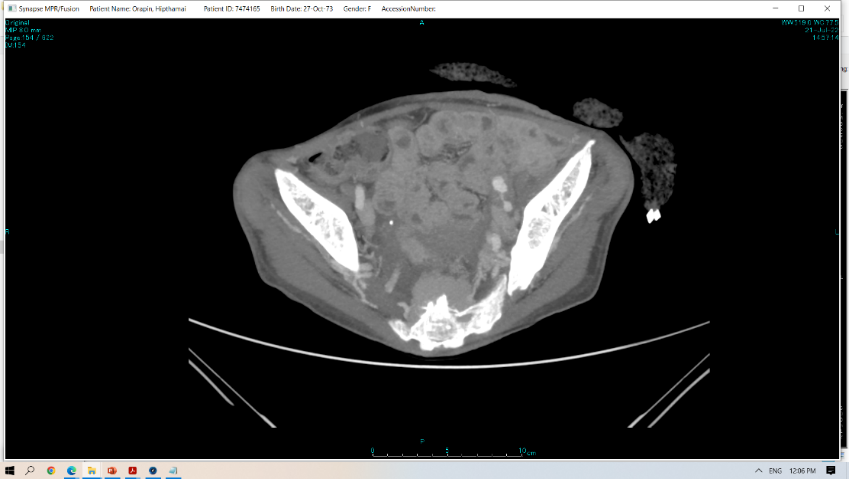
A 40-year-old female patient has been diagnosed with vascular pythiosis in the left leg. The patient initially experienced gradual left leg pain, which worsened over the course of one day before coming to the hospital. Upon examination, the patient had swelling and increased pain in the left thigh, accompanied by a cold, pale left leg. A physical examination revealed a pulseless, poikilothermic, and pale left lower extremity. The examining physician ordered a CTA of the lower extremities. Which radiological finding should be expected in this patient?

a.


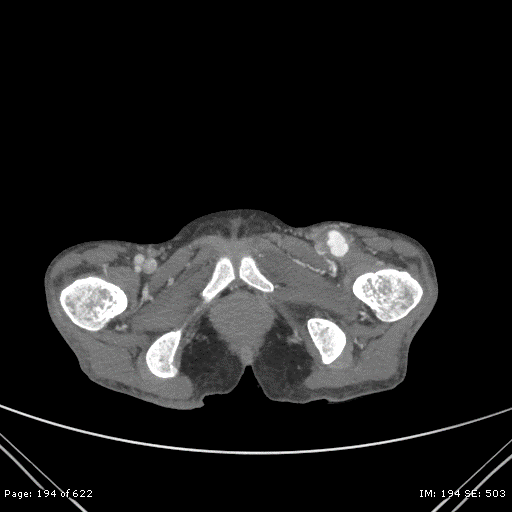


b.


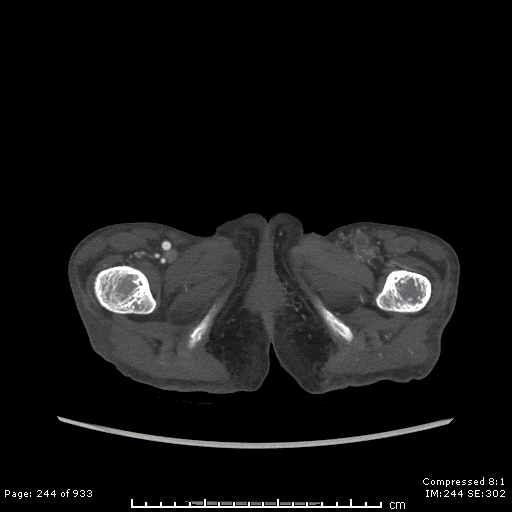


c.


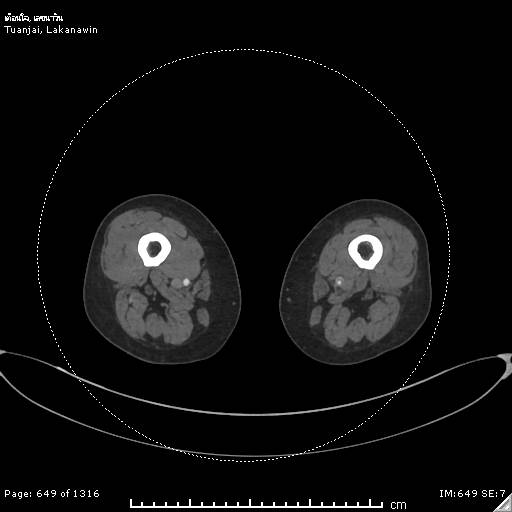


d.

1. Which of the following is the most benefit in survival rate in treatment for vascular pythiosis?

a. Antiviral medication b. Antifungal medication c. Vaccination
 d. Antibacterial medication e. Surgical removal of the infected area

1. In which drug combination is it found to enhance the synergistic effect in Pythiosis insidiosum treatment

a. Itraconazole + Terbinafine b. Voriconazole + Micafungin

c. Amphotericin + Micafungin d. Voriconazole + Terbinafine

e. Azithromycin + Doxycycline

1. A 50-year-old male patient was diagnosed with vascular pythiosis at right femoral artery three months ago and has been treated with surgery and antifungal medication. Recent tests showed no remaining infection, but the patient still experiences persistent pain and fever at the surgical site. The physician suspects an ongoing infection. Which laboratory test should be ordered to diagnose the disease?
   a. CT Angiogram b. Fungal culture c. Blood culture
   d. X-rays of the surgical site e. Galactomannan assay
2. A 46-year-old female patient was diagnosed with vascular pythiosis at right popliteal artery and has undergone surgery and antifungal treatment. Recent tests showed no remaining infection, but the patient still experiences persistent pain at the surgical site, and the beta-d-glucan level remains elevated (>500 pg/ml). Further examination revealed signs of blood clot formation in the right femoral artery. Which treatment approach would increase survival for this patient?

a. Adding micafungin to the current medication
b. Adding amphotericin to the current medication
c. Additional surgical removal of the infected area
d. Initiating vaccination against Pythium insidiosum
e. Adding vancomycin and meropenem to the current medication

**Part 3: preference on continuing professional development**

1. Which training format are you most interested in? (Divided by time)

a. synchronous learning b. asynchronous learning
c. A combination of the above two formats

1. Which training format are you most interested in? (Divided by location)
   a. onsite (in-person classroom) b. online (web-based)
   c. Hybrid training (combination of in-person and online)
2. When did you first become acquaintance with vascular pythiosis?
   a. Before becoming a medical student
   b. While being a medical student
   c. While being an intern
   d. While being a resident
   e. Never heard of it before
3. How important do you think knowledge about vascular pythiosis is for patient care?
   a. Very important b. Important c. Not important
4. Please specify the ideal duration for learning about vascular pythiosis.

a. Less than 30 minutes b. 30 minutes to 1 hour
c. More than 1 hour to 1 hour 30 minutes
d. More than 1 hour 30 minutes to 2 hours
e. More than 2 hours

1. If there were free training on vascular pythiosis, would you enroll?

A. Yes B. No C. Unsure

1. Please provide any additional suggestions for developing vascular pythiosis training. (You can leave your suggestions here)
   …………………………………………………………………………………………………………………………….

…………………………………………………………………………………………………………………………….

…………………………………………………………………………………………………………………………….

…………………………………………………………………………………………………………………………….
