# Supplementary material for: Needs assessment of a pythiosis continuing professional development program
Source: PLoS Negl Trop Dis. 2024 Feb 26;18(2):e0012004. doi: 10.1371/journal.pntd.0012004 (PMC10919846; doi:10.1371/journal.pntd.0012004)
Supplement: S2 Supplement — Table of specification of test items. (DOCX) [file pntd.0012004.s002.docx]

**Supplement 2: table of specification of test items**

| **Learning objectives: By the end of activity, ≥ 50% of participants can…** | **Number of items** |
| --- | --- |
| 1. Identify at least 1 endemic area of vascular Pythiosis | 2 |
| 2. Identify at least 1 risk factor of vascular Pythiosis | 2 |
| 3. List at least 1 sign of vascular Pythiosis | 2 |
| 4. List at least 1 symptom of vascular Pythiosis | 2 |
| 5. Identify at least 1 microbiological characteristic of vascular Pythiosis | 2 |
| 6. Identify at least 1 microbiological investigation of choice of vascular Pythiosis | 2 |
| 7. Identify at least 1 radiographic investigation of choice of vascular Pythiosis | 2 |
| 8. Interpret correctly more than 50% in a radiographic investigation of vascular Pythiosis | 2 |
| 9. Identify at least 1 treatment of vascular Pythiosis | 4 |
