# Supplementary material for: Needs assessment of a pythiosis continuing professional development program
Source: PLoS Negl Trop Dis. 2024 Feb 26;18(2):e0012004. doi: 10.1371/journal.pntd.0012004 (PMC10919846; doi:10.1371/journal.pntd.0012004)
Supplement: Attachment — Submitted filename: 240102_Reviewer comments.docx [file pntd.0012004.s004.docx]

First of all, I would like to congratulate the authors for finishing this work. I enjoy reading this manuscript. It gives important information in Continuing Professional Development (CPD) about pythiosis. This kind of work in pythiosis has never been conducted before. The result of this work provides essential information for setting up an effective and efficient learning program for clinicians. Therefore, hopefully, the awareness of the clinicians in pythiosis is increased, the prompt treatment can be given earlier, to reduce the the mortality and morbidity of pythiosis cases.

I have only a few comments:

- In the study population. Why were they only the internal medicine residents and surgery residents? Why were the General Practitioners (GPs) not included? In my opinion, the awareness of GPs about pythiosis is important for early diagnosis, since they can refer to the specialist before the condition gets worse.
- Line 143: check again the other fungal infections that mimic pythiosis, and add references.
