# Supplementary material for: Needs assessment of a pythiosis continuing professional development program
Source: PLoS Negl Trop Dis. 2024 Feb 26;18(2):e0012004. doi: 10.1371/journal.pntd.0012004 (PMC10919846; doi:10.1371/journal.pntd.0012004)
Supplement: Attachment — Submitted filename: PNTD-D-23-01592 Response to reviewers comments-cover letter.docx [file pntd.0012004.s005.docx]

February 15^th^, 2024

## Re: PNTD-D-23-01592

Dear Prof. Joshua Nosanchuk and Prof. Paul Brindley,

Editors of *PLOS Neglected Tropical Diseases*:

We appreciate you and the reviewers for your precious time reviewing our paper and providing valuable comments. Your helpful and insightful comments led to possible improvements in the current version. I am writing in response to the reviewers’ comments for our research manuscript entitled "A Needs Assessment for the Development of a Continuing Professional Development Program on Pythiosis" for consideration for publication in *PLOS Neglected Tropical Diseases*.

**Author responses to Reviewer comments:**

**Reviewer 1:**

1. Methods: Methodology, populations, sample size, and statistics were all clearly established. There is no concern about the ethical issue.

**Author response:** Thank you for the kind comment, we appreciate it.

2. Results: Everything seems OK to follow.

**Author response:** Thank you for the kind comment, we appreciate it.

3. Conclusions: Everything seems OK to follow.

**Author response:** Thank you for the kind comment, we appreciate it.

4. Summary and General Comments: Leksuwankun et al. conducted a questionnaire-based research to survey knowledge of pythiosis in Thailand among residency trainees in King Chulalongkorn Memorial Hospital. A small sample size limited the study, and some issues need to be addressed point-by-point as follows:

The authors stated that vascular pythiosis is uncommon and challenging to diagnose due to similar clinical presentations to other fungal infections such as mucormycosis, talaromycosis, and aspergillosis. This infection is usually underdiagnosed due to several factors, such as slow progress or gradual onset of vascular invasion, patient and physician under-recognition, and rarity of the disease.

**Author response:** Thank you for this comment. We have added the following on lines 144-145: “in addition to other factors such as the gradual onset of vascular invasion, patient and physician under-recognition, and disease rarity.”

5. Primary physicians, family doctors, or community doctors are additional focused groups to develop CPD to enhance early diagnosis of pythiosis and referral. According to the rarity but high disease burdens, early recognition and diagnosis should effectively reduce this threat.

**Author response:** Thank you for this comment. We have added the following on lines 357-359: “Further studies should investigate a broader range of participants including general practitioners, family medicine clinicians, and community clinicians…”

6. Proportions of PGY from Y1-Y5 were too different; this may affect the performance assessment.

**Author response:** Thank you for highlighting this point. We have included the following on lines 219-221 and 356-357 to address this: “The internal medicine residency is a three-year program, and the surgery residency is a five-year program with a lower number of residents in the fourth and fifth years.” and “Moreover, the varying proportion of senior and junior residents might affect the performance outcomes.”

7. TB exposure in the educational program is much more common in Thailand. Meanwhile, in the case of rare diseases, other examples of developing physicians' diagnosis performance, such as infrequent diseases, should be reviewed and discussed.

**Author response:** We appreciate your emphasis on this point. As far as we know, this is the first educational research endeavor focusing on a rare and fatal disease in Thailand. To address this, we included the following on lines 283-285: “To our knowledge, this is the first educational program focused on a rare infectious disease in Thailand.”

8. What are the root causes of underscore in vascular pythiosis lab investigations, symptoms, and epidemiology among residents? This point is intriguing to discuss.

**Author response:** Thank you for your suggestion. We have included the following on lines 329-330: “The low scores could be explained by the lack of exposure in each domain, attributed to the disease rarity.”

9. Another future research and development would be increasing awareness of this invasive disease, particularly in high-risk populations.

**Author response:** We agree with your comment. We have included the following on lines 359-360: “increasing awareness of pythiosis (particularly in high-risk populations)”

**Reviewer 2:**

None

**Reviewer 3:**

1. Methods: The authors conducted a needs assessment survey and obtained a good survey completion rate of 67%. The study objectives were clearly articulated and was appropriate to address stated objectives. Based on the author's statistical analysis it appears the sample size was adequate. The sampling technique was voluntary sampling – it’s possible that residents with some knowledge of or interest in pythiosis were more likely to take the survey which might have resulted in an over-estimation of knowledge of pythiosis in the sample group.

**Author response:** Thank you for this comment. We have added the following sentence on lines 353-356: “Additionally, the sampling technique was voluntary sampling – it is possible that residents with prior knowledge or interest in pythiosis were more likely to participate in the study, which may have resulted in an over-estimation of pythiosis knowledge in the study group.”

2. Results: Analysis presented matches analysis plan and results were clearly presented.

Tables and figures were appropriate and easy to understand.

**Author response:** Thank you for the kind comment, we appreciate it.

3. Conclusions: The author's conclusions are appropriate given the survey findings. An educational program as proposed by the authors would likely only be applicable in regions such as South and Southeast Asia as the disease is extremely uncommon in the rest of the world. It could be generalizable though to infectious disease specialists even outside of these regions.

**Author response:** Thank you for this comment. We agree that this program is applicable and generalizable to specialists and generalists outside of these regions. We feel that this statement doesn’t need to be explicitly included in the manuscript; however, if you wish for us to include this, we are happy to.

4. Line 133-134 - Assertion that all vascular pythiosis cases are reported from Thailand is incorrect and needs to be corrected. See Travel Med Infect Dis. 2022 Jul-Aug:48:102349. by Perkins et al. in which two human cases of vascular pythiosis from North America are reported (one acquired in Jamaica, one acquired in Texas).

**Author response:** Thank you for this correction, we agree with the reviewer and changed the language from all cases to “most of” the cases on line 133.

5. Line 328 – Pythiosis occurs outside of India and Thailand. Authors should change this sentence to something like “pythiosis is most prevalent in India and Thailand.”

**Author response:** Thank you for bringing this to our attention. We made the change on line 334 to say: “is most prevalent in India and Thailand”

6. Line 128 - would change “low prevalent” to low-prevalence

**Author response:** Thank you for the suggestion. We made the change on line 128 to say: “low-prevalence”

7. Line 291 - in the U.S. we usually use the term “infection control” instead of “infectious control” and the authors could consider changing this.

**Author response:** Thank you for the suggestion. We have made the changes on lines 296 to say: “infection control”

8. Line 304 – see above comment.

**Author response:** Thank you for the suggestion. We have made the changes on lines 309 to say: “infection control”

9. This paper addresses an important aspect this this rare infection – lack of knowledge of pythiosis by health care providers. As mentioned in the paper, time to diagnosis is an important factor in the efficacy of subsequent therapy so an educational program, such as that suggested by the authors, could be very important in improving outcomes of patients with this rare disease. Education of providers on the subject of pythiosis has rarely, if ever, been addressed in the medical literature and this paper is an important contribution to the literature on pythiosis.

**Author response:** Thank you for the kind comment, we appreciate it.

**Additional reviewer comments (from attached word document):**

1. First of all, I would like to congratulate the authors for finishing this work. I enjoy reading this manuscript. It gives important information in Continuing Professional Development (CPD) about pythiosis. This kind of work in pythiosis has never been conducted before. The result of this work provides essential information for setting up an effective and efficient learning program for clinicians. Therefore, hopefully, the awareness of the clinicians in pythiosis is increased, the prompt treatment can be given earlier, to reduce the the mortality and morbidity of pythiosis cases.

**Author response:** Thank you for the kind comment, we appreciate it.

2. In the study population. Why were they only the internal medicine residents and surgery residents? Why were the General Practitioners (GPs) not included? In my opinion, the awareness of GPs about pythiosis is important for early diagnosis, since they can refer to the specialist before the condition gets worse.

**Author response:** Thank you for your suggestion. We concur that including general practitioners as frontline healthcare providers is imperative; however, due to feasibility, we were unable to include them at this time. We have acknowledged this limitation in lines 347-351: “First, the participants in this study were internal medicine and surgery residents at a single institution due to feasibility. This selection does not encompass all physicians in Thailand, particularly the general practitioners who serve as frontline healthcare providers. Nevertheless, based on this study, we can infer that there may be a knowledge gap among general practitioners as well.”

3. Line 143 - please review the other fungal infections that mimic pythiosis and include references in the introduction section.

**Author response:** Thank you for your suggestion. We have corrected "vascular pythiosis" to "human pythiosis" on line 141, which is more accurate. Additionally, we have included reference 9 on line 144.

**Other modifications to the manuscript according to technical check requests:**

1. Data availability statement has been changed from “Data presented in this study is available on request from the corresponding author.” to “Data presented in this study is available and freely accessible from the supporting information.” on lines 379-380.

2. The list of legends of supporting information files has been added after the references list on lines 467-471.

3. A statement that formal consent was not obtained has been added as “Therefore, formal written consent was not obtained due to anonymity” on lines 186-887.

Thank you for your time and consideration. We look forward to your response and the opportunity to contribute to the scientific community through the pages of *PLOS Neglected Tropical Diseases*.

Sincerely yours,

Rongpong Plongla, MD, MSc, D(ABMM)

Corresponding author:

Rongpong Plongla, MD, MSc, D(ABMM)

E-mail: rongpong.p@chula.ac.th, rongpong@hotmail.com

Address: 1873 Rama IV Rd, Pathumwan, Bangkok, Thailand 10330
Tel: 66-2256-4000 EXT 80506

Alternate corresponding author:

Surachai Leksuwankun, MD, MSc (HSE)

E-mail: surachaileksuwankun2@gmail.com

Address: 1873 Rama IV Rd, Pathumwan, Bangkok, Thailand 10330

Tel: 66-2256-4000 EXT 80506
